# Polynomial fitting method of background correction for electron backscatter diffraction patterns

**DOI:** 10.1038/s41598-021-04407-0

**Published:** 2022-01-10

**Authors:** Yi-Yun Tsai, Yi-Chen Pan, Jui-Chao Kuo

**Affiliations:** Department of Materials Science and Engineering, National Cheng-Kung University, Tainan, 701 Taiwan

**Keywords:** Characterization and analytical techniques, Microscopy

## Abstract

A raw electron backscatter diffraction (EBSD) signal can be empirically decomposed into a Kikuchi diffraction pattern and a smooth background. For pattern indexing, the latter is generally undesirable but can reveal topographical, compositional, or diffraction contrast. In this study, we proposed a new background correction method using polynomial fitting (PF) algorithm to obtain clear Kikuchi diffraction patterns for some applications in nonconductive materials due to coating problems, at low accelerated voltage and at rough sample surfaces and for the requirement of high pattern quality in HR-EBSD. To evaluate the quality metrics of the Kikuchi patterns, we initially used three indices, namely, pattern quality, Tenengrad variance, and spatial–spectral entropy-based quality to detect the clarity, contrast, and noise of Kikuchi patterns obtained at 5 and 15 kV. Then, we examined the performance of PF method by comparing it with pattern averaging and Fourier transform-based methods. Finally, this PF background correction is demonstrated to extract the background images from the blurred diffraction patterns of EBSD measurements at low kV accelerating voltage and with coating layer, and to provide clear Kikuchi patterns successfully.

## Introduction

Recently, electron backscatter diffraction (EBSD) in scanning electron microscopy (SEM) has become a standard analysis technique in materials science because of increasing requirements of its application in ultrafine-grained nanocrystalline materials^1–3^. However, the application of the EBSD technique is limited by its lateral and longitudinal resolution, which can be improved by reducing the accelerating voltage, applying an energy filter^4,5^, and decreasing the thickness of specimens using t-EBSD^6,7^. Steinmetz and Zaefferer measured the physical lateral resolution of 30 nm and 10 nm for twinning-induced plasticity steel samples at accelerating voltages of 15 and 7.5 kV, respectively^8^. Chen et al.^9^ reported that the best physical lateral resolution of 34.5 nm is obtained at an accelerating voltage of 10 kV for copper. Tripathi and Zaefferer^10^ obtained the best lateral resolution of 240 nm at an accelerating voltage of 5 kV for magnesium. Moreover, the reduction of the accelerating voltage is accompanied by disadvantages, such as reduced source brightness, increased chromatic aberration, increased sensitivity to stray fields, and deflection of the beam from the secondary electron collection field^9^. Furthermore, a decrease in the accelerating voltage decreases the total number of backscattered electrons and the electrons detected by the CCD detector^9^. Fortunately, EBSD measurements at low accelerating voltages have extraordinary advantages of limiting the charge in semi- or nonconducting samples^10,11^, reducing the electron–beam interaction volume in high spatial resolution mapping^12,13^, and capturing enhanced band contrast, higher-order bands, or inelastic scattering effects^14,15^.

Kikuchi patterns are formed by near-surface electrons having energy loss within 10% of the incident beam energy, called low-loss electrons^6,16^. The formation of backscatter Kikuchi patterns can be explained by the formation of incoherent point sources within a material and then by the subsequent elastic scattering. During the consecutive scattering events, occur single or multiple inelastic and elastic scattering occurs, leading to incoherent point sources within a material^10,17,18^. Consequently, electrons from these point sources experience elastic scattering at given Bragg angles to form Kikuchi bands, whereas the other electrons do not undergo elastic scattering at given Bragg angles to contribute to diffuse background^3,4,17,19^. Winkelmann et al.^20^ and Deal et al.^21^ stated that the influence factors on the background intensity distribution in EBSD patterns include the accelerating voltage of the incident beam and the pattern center of EBSD patterns, as well as the working distance and the tilt angle.

EBSD patterns can be empirically divided into two partitions of Kikuchi diffraction (KD) and background (BG)^22–25^. Then, the background correction method is generally used for the post-processing of EBSD patterns to solve the problem of low signal-to-noise ratio in EBSD patterns. Here, the background division and subtraction for the background correction method are used for multiplicative and additive processing of images, respectively. However, the static background division is usually applied for the image post-processing of Kikuchi patterns, that is, to generate a background through averaging Kikuchi patterns obtained from many differently oriented grains^26^. In addition, Winkelmann^15^ proposed a dynamic correction method, which generates a background for each individual pattern, and Britton et al.^27^ reported a background construction method using a Gaussian model approach.

In the present study, we propose a new polynomial fitting (PF) background correction method for correcting the background in EBSD patterns and providing high-quality Kikuchi patterns. This method combines the concepts of the dynamic background correction and the static background correction. The potential applications can be found in nonconductive materials, at low accelerated voltage and at rough sample surfaces, and for the requirement of high pattern quality in HR-EBSD reported by Wilkinson^28^. Furthermore, we compare this method with two background correction methods using pattern averaging (PA) and Fourier transform-based (FT) approaches. In addition, to evaluate the quality metrics of the Kikuchi patterns prior to indexing, we use three techniques, namely, pattern quality (PQ), Tenengrad variance (TenV), and spatial–spectral entropy-based quality (SSEQ) to detect the clarity, contrast, and noise of processed images, respectively.

## Results and discussion

### Evaluation of quality metrics of Kikuchi patterns

In the nonconductive material applications, at low accelerated voltage and at rough sample surfaces, EBSD measurements show blurred patterns. The quality of Kikuchi patterns has a significant influence on the performance of the indexing processing. Thus, the quality metrics should be applied to evaluate the image quality. Three techniques, namely, PQ, TenV, and SSEQ are applied to quantify the quality of images with respect to contrast, blur, and noise. First, the definition of PQ is presented as follows^29,30^:

1 $$PQ=\frac{1}{N}\sum_{i=1}^{N}H\left({\rho }_{i}, {\theta }_{i}\right),$$

where *N* is the number of diffraction bands being five, and *H* is the height of the Hough peak corresponding to a band at location $\left({\rho }_{i}, {\theta }_{i}\right)$. Here, PQ is used to detect the sharpness of the observed bands, and TenV and SSEQ are used to the image quality.

Second, TenV^31^, which represents the gradient magnitude between pixels in an image, is given by the following:

2 $$TenV\left(I\right)=\sum_{m=1}^{M}\sum_{n=1}^{N}[S{\left(m,n\right)-\overline{S }]}^{2} \overline{S }=\frac{1}{NM}\sum_{m=1}^{M}\sum_{n=1}^{N}S\left(m,n\right),$$

where $S\left(m,n\right)$ represents a gradient magnitude of $I\left(m,n\right)$ pixels, and $\overline{S }$ is the mean of the magnitudes given by the following:

3 $$\overline{S }=\frac{1}{NM}\sum_{m=1}^{M}\sum_{n=1}^{N}S\left(m,n\right).$$

The larger deviation of the gray level indicates the higher the TenV value.

Third, SSEQ is a type of no-reference image quality assessment (called NR-IQA) approach to evaluate the degree of distortion in a single image; it is based on the calculation of spectral and spatial entropy^32^. The SSEQ value from 0 to 100 indicates good to poor quality. The procedure of SSEQ approach can be roughly divided into four steps, as follows: down sampling process, partition image to $M\times M$ blocks, feature pooling, and prediction of the image quality score. The spatial entropy is given by the following:

4 $${E}_{s}=-\sum_{x}p\left(x\right){log}_{2}p\left(x\right)$$

where *x* is the pixel value within a block, with empirical probability density $p\left(x\right)$, i.e., relative frequency here. The spectral entropy is given by the following:

5 $${E}_{f}=-\sum_{i}\sum_{j}P(i,j){log}_{2}P(i,j)$$

where $P(i,j)$ is a spectral probability map.

**Figure 1:**
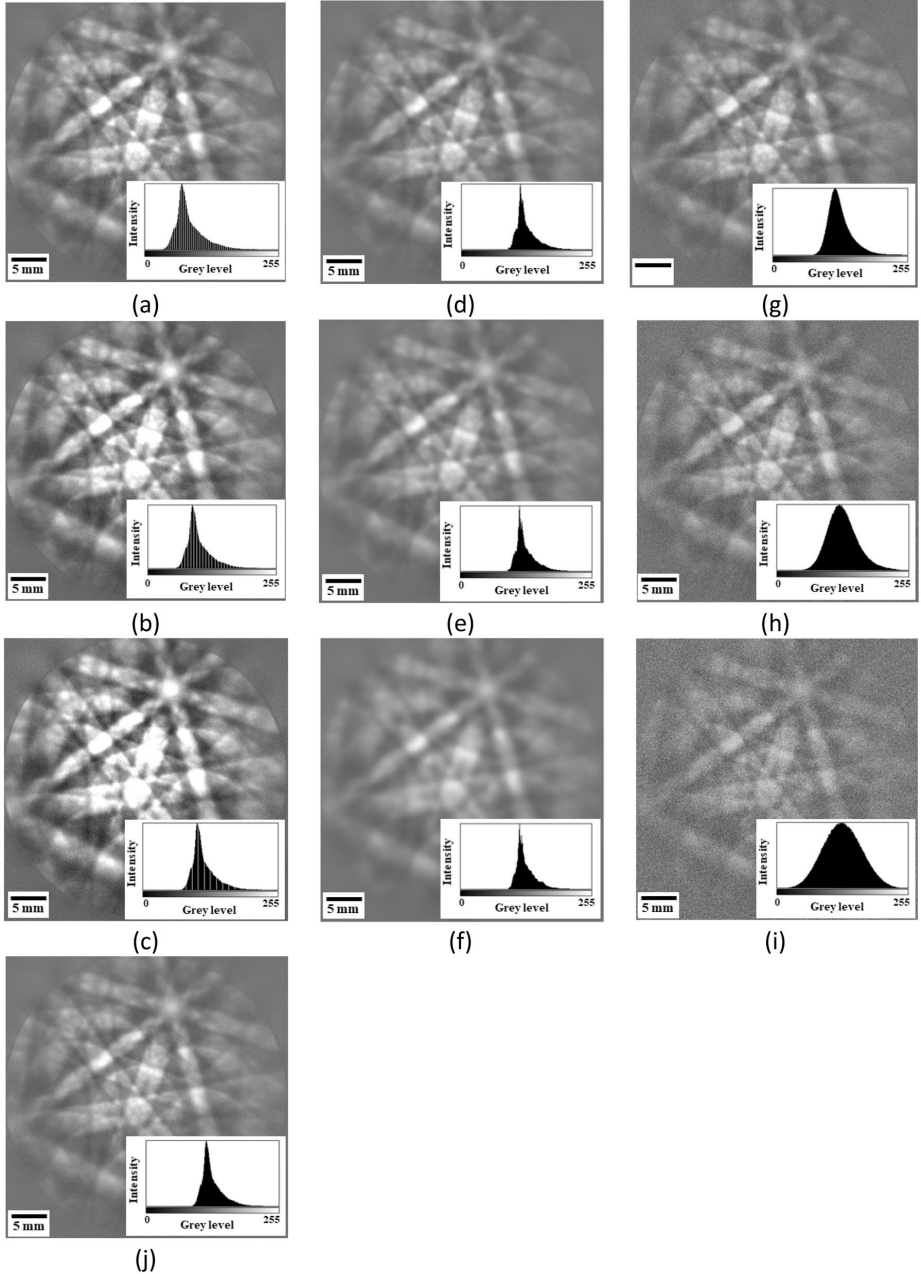
Contrast-modified images with the low gray level of (**a**) 63, (**b**) 42 and (**c**) 21; sharpness-modified images using Gaussian blur filter with the standard deviation ${\sigma }_{1}$ of (**d**) 3, (**e**) 5 and (**f**) 10; and noise-modified images using Gaussian noise with the standard deviation ${\sigma }_{2}$ of (**g**) 15, (**h**) 35 and (**i**) 70 with respect to (**j**) raw image as the reference image.

After introduction to three image quality metrics of PQ, TenV, and SSEQ, modifications of contrast, sharpness, and noise were carried out on a raw image in Fig. 1j. For contrast modification, the low limit of the gray level is selected at 21, 42, and 63 to fix the gray level at 255. Figure 1a–c show that the low limit from 63 to 21 increases the image contrast because of the increasing range of the gray level^33^. When considering the increasing percentage with respect to the raw image, that of TenV is significantly larger than that of PQ, whereas SSEQ shows slightly decreasing percentage. Thus, TenV and PQ metrics are sensitive to the change in image contrast in Table 1.

**Table 1:** Indexing values of PQ, TenV, and SSEQ after modification of contrast, blur and noise.

**Contrast adjustment**				
Gray level	–*	63–255	42–255	21–255
PQ	10,024	19,264	24,519	26,075
TenV	7.02 × 10^5^	2.42 × 10^6^	6.55 × 10^6^	1.48 × 10^7^
SSEQ	13.03	9.89	10.69	11.82
**Blur adjustment**				
${\sigma }_{1}$	–*	3	5	10
PQ	10,024	9923	7728	7190
TenV	7.02 × 10^5^	4.02 × 10^4^	2.39 × 10^4^	6.43 × 10^3^
SSEQ	13.03	32.11	64.01	67.51
**Noise adjustment**				
${\sigma }_{2}$	–*	15	35	70
PQ	10,024	9924	7729	7191
TenV	7.02 × 10^5^	3.46 × 10^7^	8.58 × 10^8^	6.01 × 10^9^
SSEQ	13.03	41.01	52.94	61.09

*Remark: without any adjustment.

In the case of sharpness modification, a 2D Gaussian blur filter (G_2D_) called a nonuniform low-pass filter that preserves the low spatial frequency and smooth images is expressed as follows^34^:

6 $${G}_{2D}=\left(x,y,{\sigma }_{1}\right)=\frac{1}{{2\pi \sigma }^{2}}{e}^{-\frac{{x}^{2}+{y}^{2}}{2{{\sigma }_{1}}^{2}}},$$

where *x* and *y* are the location positions. The standard deviation ${\sigma }_{1}$ of the Gaussian blur filter changes the amount of blurring. When increasing the standard deviation ${\sigma }_{1}$ from 3 to 10, the images become more blurred, as shown in Fig. 1d–f. The increase in the standard deviation from 3 to 10 decreases the PQ value from 9923 to 7190, but increases TenV value from 4.02 $\times$ 10^4^ to 6.43 $\times$ 10^3^ and the SSEQ value from 32.11 to 67.51, as shown in Table 1. When considering the increasing percentage with respect to the raw image, the indexing of TenV is observed to be more sensitive than that of SSEQ due to the sharpness change, but that of PQ is insensitive.

When increasing noise in images, the probability density function called Gaussian noise^35^ of a random Gaussian variable *z* is given by the following:

7 $$PG\left(z\right)=\frac{1}{{\sigma }_{2}\sqrt{2\pi }}{e}^{-\frac{{\left(z-\mu \right)}^{2}}{2{{\sigma }_{2}}^{2}}},$$

where *z* represents the gray level, $\mu$ is the mean gray value, and ${\sigma }_{2}$ is the standard deviation. When increasing standard deviation ${\sigma }_{2}$ from 15 to 70, the PG value becomes larger, indicating the broader distribution of noise in Fig. 1g–i. The increase in the standard deviation from 15 to 70 decreases the PQ value from 9924 to 7191 and TenV value from 3.46 $\times$ 10^7^ to 6.01 $\times$ 10^9^, but increases the SSEQ value from 41.01 to 61.09, as shown in Table 1. Although the indexing of TenV shows a more increasing percentage here, the increasing TenV value indicates a sharp image, which is inappropriate. Here, the indexing of SSEQ is more sensitive than that of PQ to detect noise. Therefore, the TenV indexing is sensitive to change in contrast and blur and the SSEQ indexing to noise detection.

### Estimation of background image by PA, FT, and PF methods

**Figure 2:**
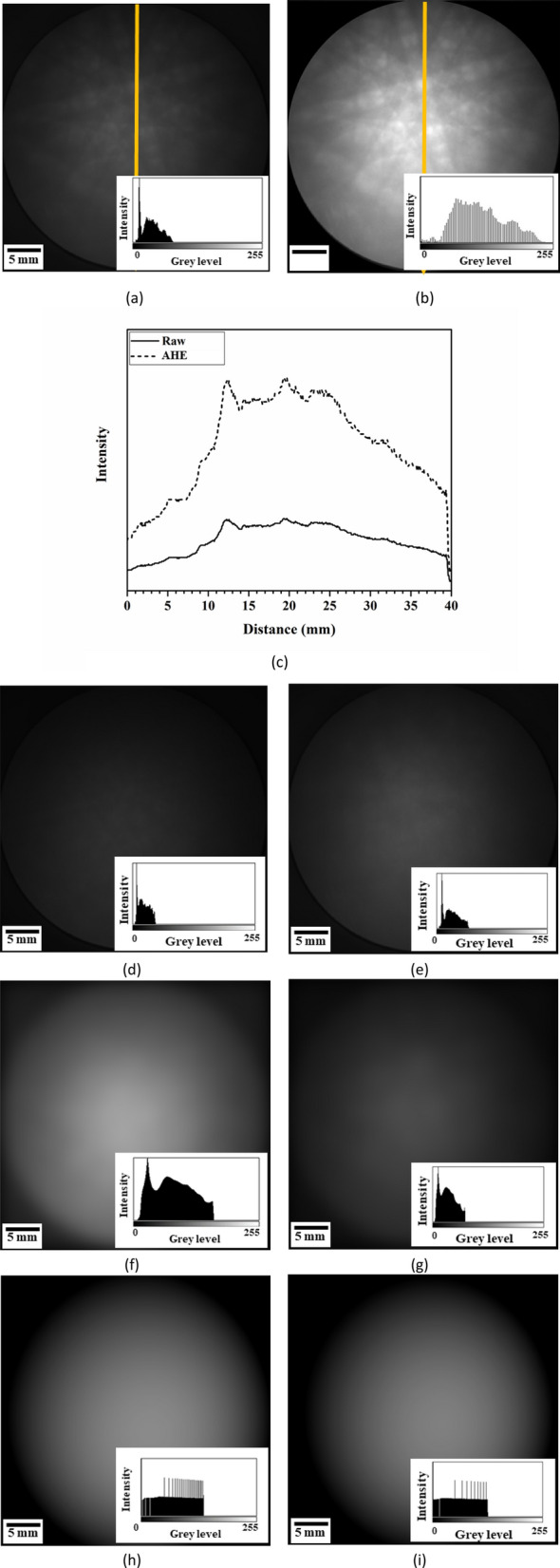
(**a**) Raw image, (**b**) contrast-enhanced image using AHE filter at 5 kV, and (**c**) the gray-level profile comparison between raw and contrast-enhanced images. Background generation by (**d**,**e**) PA, (**f**,**g**) FT, and (**h**,**i**) PF methods, where (**d**,**f**,**h**) are for 15 kV and (**e**,**g**,**i**) are 5 kV.

At low accelerated voltage, Kikuchi patterns reveal blurred edges with a low signal-to-noise ratio. Thus, adaptive histogram equalization (AHE) is initially used to improve contrast in images. After contrast enhancement with AHE, the comparison of the raw images in Fig. 2a,b shows an improved contrast, and the enhanced contrast broadens the range of gray level in Fig. 2c. The profile of gray level marked by the yellow line is plotted in Fig. 2a,b, and the profile is significantly increased after implantation of AHE.

Diffraction patterns contain a significant amount of background, which can be approximated by averaging patterns from various crystallites in a given polycrystalline sample or simulated patterns at low magnifications^5^. This process describes the first background generation method called PA, where an average pattern is revealed without crystallographic details that remove long-range intensity gradients.

In the second method called FT^36^, a low-pass filtering operation (also called a “blurring” or “smoothing” filter) is used to selectively smooth the image background without changing the bright areas. The 2D discrete Fourier transform of an $M\times N$ image is expressed by the following:

8 $$F(u,v)=\frac{1}{MN}\sum_{x=0}^{M-1}\sum_{x=0}^{N-1}f(x,y){e}^{-j2\uppi \left(\frac{ux}{M}+\frac{vy}{N}\right)}$$

where $x$ and $y$ are the spatial variables, and $u$ and $v$ are the frequency variables. The center region of the frequency domain represents the low frequency region, which corresponds to the background signal of Kikuchi patterns. Hence, we use the Gaussian low-pass filter to remove the signal of the low frequency region. The Gaussian low-pass filter is given as follows^35^:

9 $$H\left(u,v\right)={e}^{\frac{-{D}^{2}\left(u,v\right)}{2{D}_{0}^{2}}},$$

where $D\left(u,v\right)$ is the distance from the center of the frequency rectangle, and ${D}_{0}$ is the cutoff frequency. The generated background uses the FT method, where a cutoff frequency of 10 is selected for the Gaussian low-pass filter.

In the third method, which uses the PF approach, the electron backscatter diffraction patterns are composed of a Kikuchi diffraction pattern (signal) and a smooth background (noise). To obtain a 3D surface, the 2D Kikuchi pattern should be transformed into a 3D Kikuchi curve by converting the ${g}_{i}$ value of the gray scale at (X_i_, Y_i_) to the Z_i_ value corresponding to a point at (X_i_, Y_i_, Z_i_) in 3D. The latter can be approximated by a smooth surface with polynomials^37^, which are used for curve fitting techniques and given by the following:

10 $$F\left(x,y\right)=\sum_{k=0}^{N}\sum_{i=0}^{N-k}{p}_{i,k}{x}^{i}{y}^{k}$$

where *N* is the order in the range between 1 and 5, and ${p}_{i,k}$ is the coefficient of the polynomial function. The order *N* is considered larger than 3, and the fitting background includes detailed diffraction information, that is, diffraction signals. Thus, the selected value of the order *N* is 3, which is called the poly33 model and given as follows:

11 $$Poly33=F\left(x,y\right)=p00\,+\,p10\,*\,x\,+\,p01\,*\,y\,+\,p20\,*\,{x}^{2}\,+\,p11\,*\,x\,*\,y+p02\,*\,{y}^{2}\,+\,p30\,*\,{x}^{3}\,+\,p21\,*\,{x}^{2}\,*\,y\,+\,p12\,*\,x\,*\,{y}^{2}+p03\,*\,{y}^{3}.$$

The least squares curve fitting technique is used for the solution of the best-fitting curve by minimizing the sum of the least square errors of the data points. Then, the maximum likelihood estimation for the parameter ${p}_{ij}$ can be obtained by minimizing the chi square. The optimized solution for the 3D fitting curve follows the least squares error of ${\chi }^{2}$ given by the following:

12 $${\chi }^{2}=\sum_{j=1}^{M}{\left({z}_{j}-\sum_{k=0}^{N}\sum_{i=0}^{N-k}{p}_{i,k}{x}^{i}{y}^{k}\right)}^{2},$$

13 $$\frac{\partial {\chi }^{2}}{\partial {p}_{i,k}}=0,$$

where *M* is the data number, and ${z}_{j}$ is the input value at a position of $\left({x}_{j}{,y}_{j}\right)$. The backgrounds were generated by PA, FT, and PF methods, as shown in Fig. 2d,f,h for 15 kV and in Fig. 2e,g,i for 5 kV, which are used for the subsequent background correction process.

After the polynomial regression approach, the intensity of the generated background surface can be smaller than that of the raw pattern in 3D. This observation results in a negative value after background correction. To avoid the occurrence of negative values after background correction, a $\gamma$ correction function should be used to compensate for the insufficient intensity of the generated background, and the $\gamma$ correction function is given by the following^38^:

14 $$s=c{r}^{\gamma },$$

where *r* is the gray level of the input, *s* is that of the output, and *c* is a constant. The parameters of $\gamma$ = 0.65 and c = 1 are used in $\gamma$ correction, where the $\gamma$ value is used to compensate for the insufficient intensity of the raw image.

**Figure 3:**
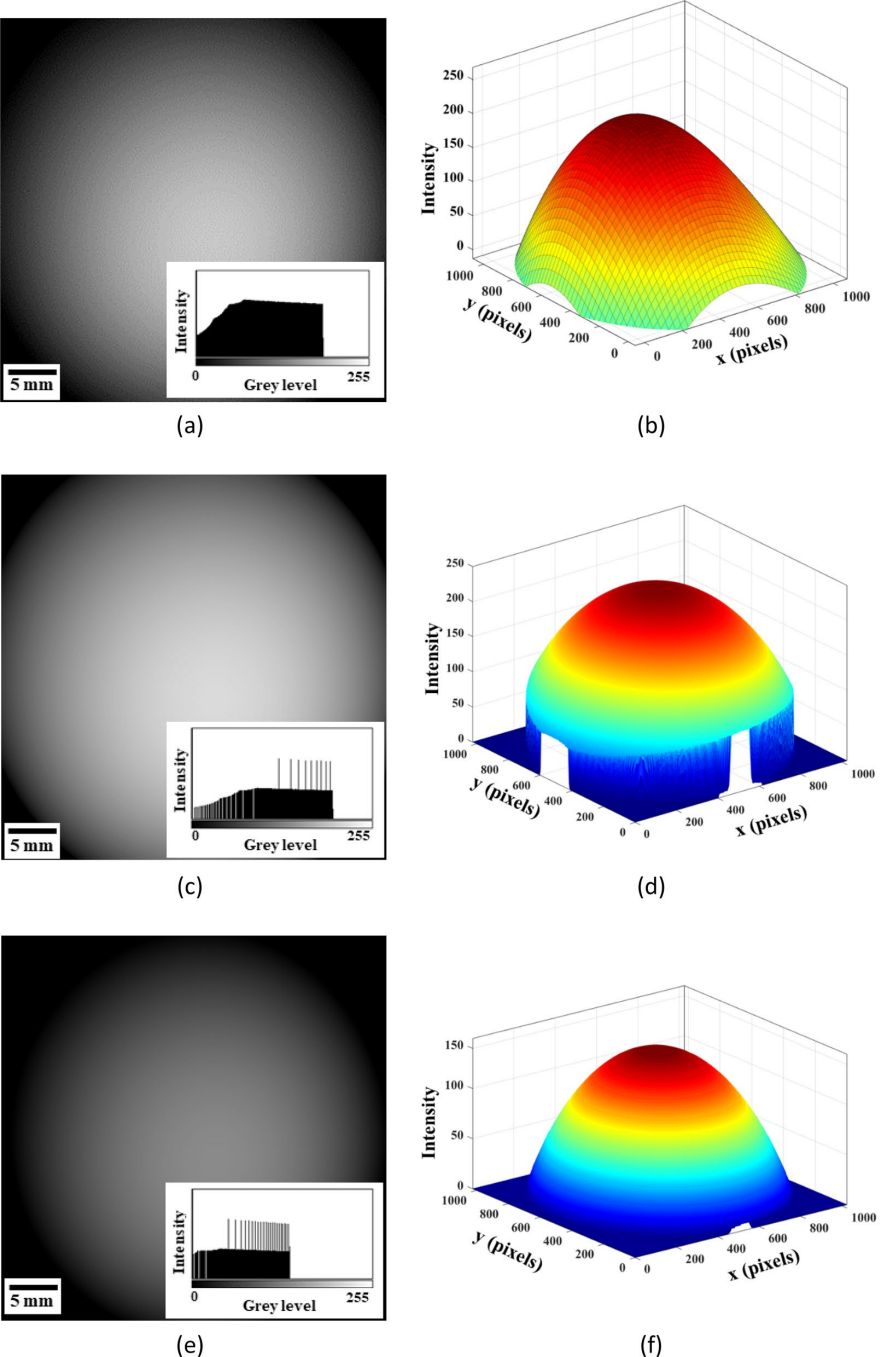
The images (**a**) without image processing, (**c**) after surface fitting and γ correction, and (**e**) after background optimization at 15 kV, where (**b**,**d**,**f**) are the 3D plots corresponding to (**a**,**c**,**e**).

**Figure 4:**
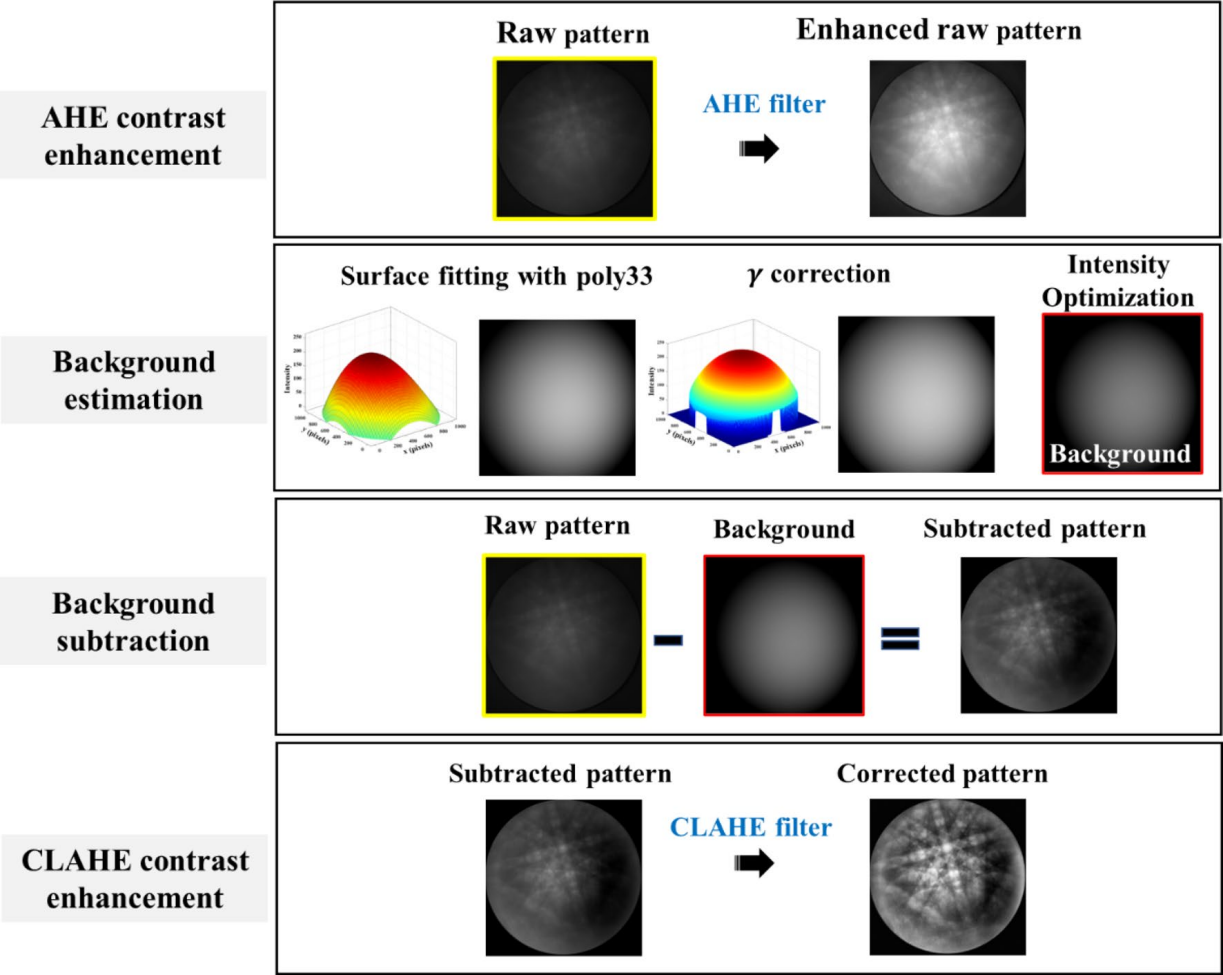
Illustration of the procedure of PF background correction algorithm.

After $\gamma$ correction and optimization processes, the generated background images in Fig. 3a,c,e reveal an improved contrast when compared with the images without $\gamma$ correction and optimization processes in Fig. 2d,f,h for 15 kV. The 3D plots of the background are shown in Fig. 3b,d,f. Thus, the background generation procedures include contrast enhancement, surface fitting, $\gamma$ correction, and background optimization, as shown in Fig. 4.

### Background correction algorithm with PF, PA, and FT methods

After hitting the specimen surface, the primary beam electrons suffer random elastic and inelastic scattering due to energy lose. After a given penetration depth, the electrons scatter at various angles and some electrons diffract following the Bragg’s law, resulting in Kikuchi bands. Thus, a relatively low-intensity signal is superimposed with a dominating background signal, as observed on the detector^39^. To solve the problem of low signal-to-noise ratio in EBSD patterns, the background correction algorithm is generally used for the post-processing of EBSD patterns. Thus, we propose a PF background correction algorithm, as shown in Fig. 4: AHE contrast enhancement, background estimation, background subtraction, and contrast-limited adaptive histogram equalization (CLAHE) contrast enhancement.

**Figure 5:**
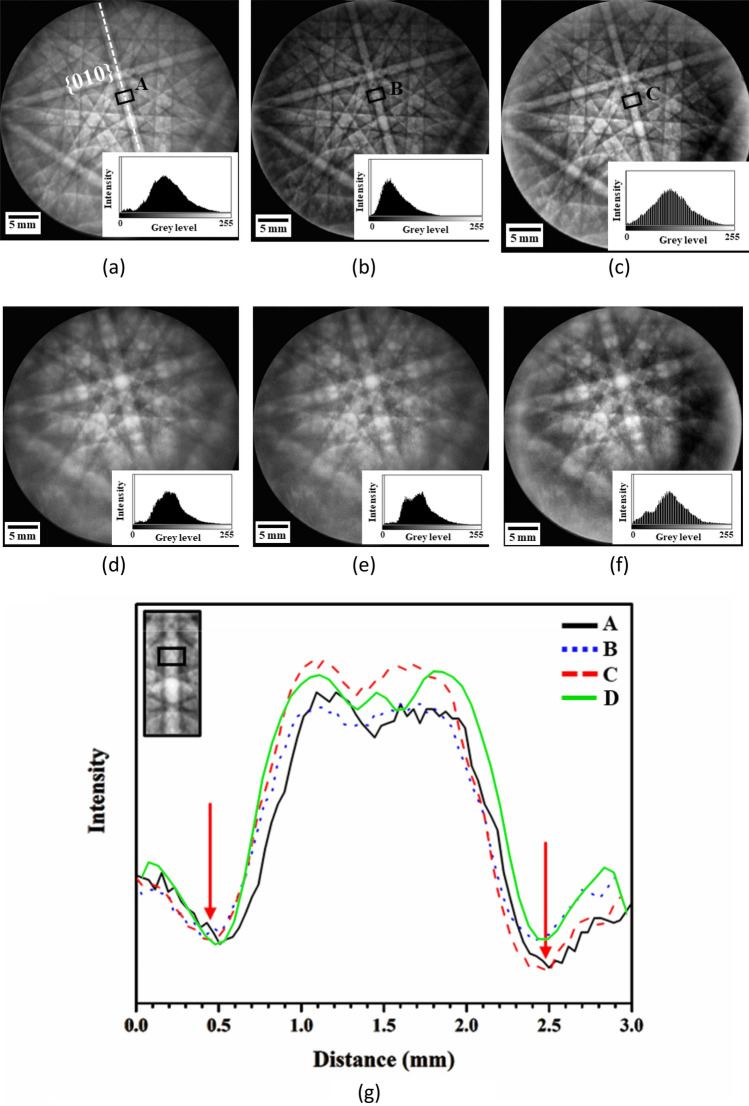
Kikuchi patterns at 15 and 5 kV after background correction by backgrounds generated by (**a**,**d**) PA, (**b**,**e**) FT, and (**c**,**f**) PF methods. (**g**) {010} Band intensity profiles of Kikuchi patterns at 15 kV after background correction using PA (A), FT (B), and PF (C) methods, where the arrow symbols show the position of the band edges detected using the PF background correction. (Green solid profile D is the simulated band profile of Ni at 15 kV).

After background subtraction, CLAHE algorithm is proposed to solve the problem of contrast degradation and to enhance the image contrast in the preprocessing stages. The CLAHE contrast enhancement is applied for PA, FT, and PF methods to compare the image quality after background subtraction, as shown in Fig. 5. The PF method at 15 kV have the highest value of the PQ and TenV indices at 18,652 and 7.33 $\times$ 10^7^, respectively, whereas the SSEQ index has the lowest value of 34.69, as shown in Table 2. These results suggest that the pattern, after the PF background correction method, reveals clear and sharp images with decreasing noise.

**Table 2:** Indexing values of PQ, TenV, and SSEQ at accelerating voltage of 15 kV and 5 kV.

kV	Index	PA	FT	PF
15 kV	PQ	12,993	10,677	18,652
	TenV	5.14 $\times$ 10^6^	2.92 $\times$ 10^7^	7.33 $\times$ 10^7^
	SSEQ	35.62	35.02	34.69
5 kV	PQ	4921	13,699	24,062
	TenV	2.49 $\times$ 10^6^	4.90 $\times$ 10^6^	3.41 $\times$ 10^7^
	SSEQ	18.54	19.68	18.92

In addition, in the case of 5 kV, PQ and TenV indices have the highest value of 24,062 and 3.41 $\times$ 10^7^, but no significant difference is observed in the SSEQ value for PF method. Here, the PF method provides a bright background image with a broad intensity distribution at 5 kV. Therefore, we successfully demonstrate that the PF background correction method in addition to PA and FT methods can effectively extract a background and Kikuchi pattern from raw diffraction patterns.

To evaluate the image quality after background correction, the profiles of $\left\{010\right\}$ band width represented by black areas called A, B, and C are shown in Fig. 5a–c for PA, FT, and PF methods, respectively. The simulated edges of the $\left\{010\right\}$ Kikuchi band in Fig. 5g are compared, and the $\left\{010\right\}$ profile of the C area for the PF method indicates a significant difference between peaks and valleys, that is, a clear band pattern for pattern indexing.

### Case study of PF background correction

The spatial resolution of EBSD can be improved at low accelerated voltage for nonconductive materials. However, lowering the beam energy of the electrons reduces the mean free paths of these elastic and inelastic processes rapidly^33^. Moreover, considering nonconductive samples, conductive coating should be required on the sample surface with a thin amorphous layer of gold or platinum to minimize charging effects^40^. Unfortunately, the effects of low accelerated voltage and conductive coating layer decrease the quality of Kikuchi patterns^12^.

**Figure 6:**
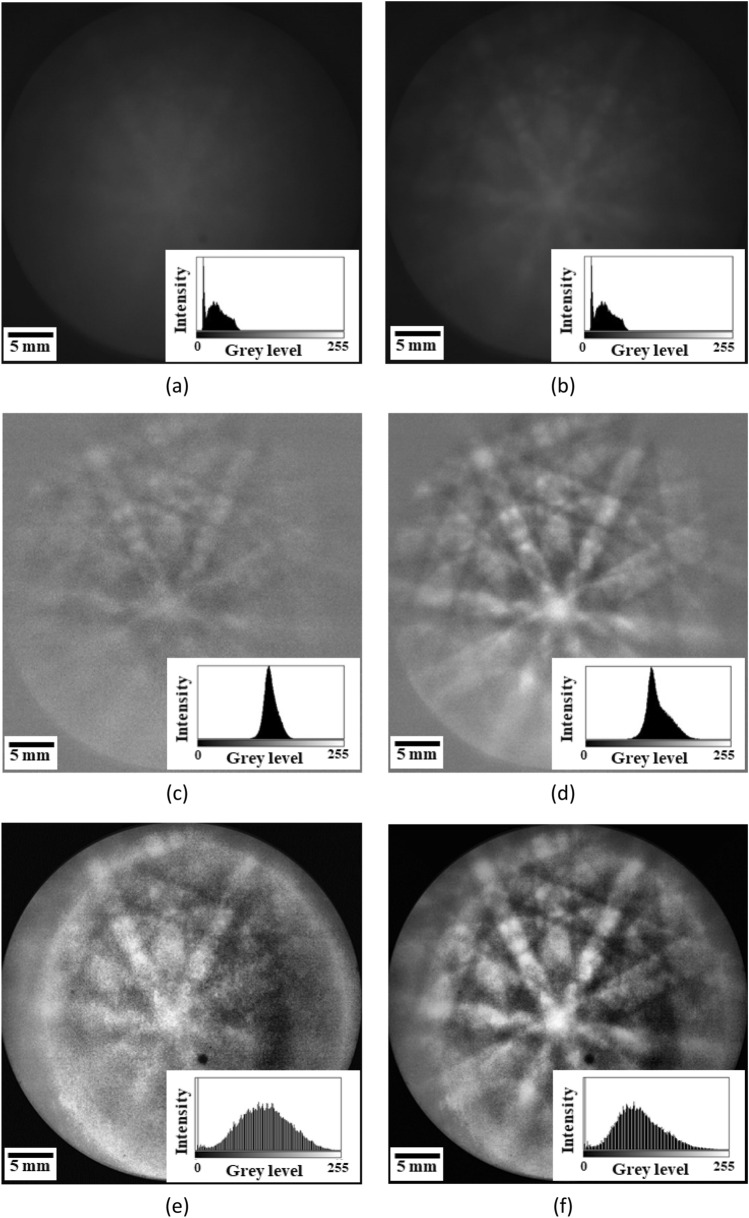

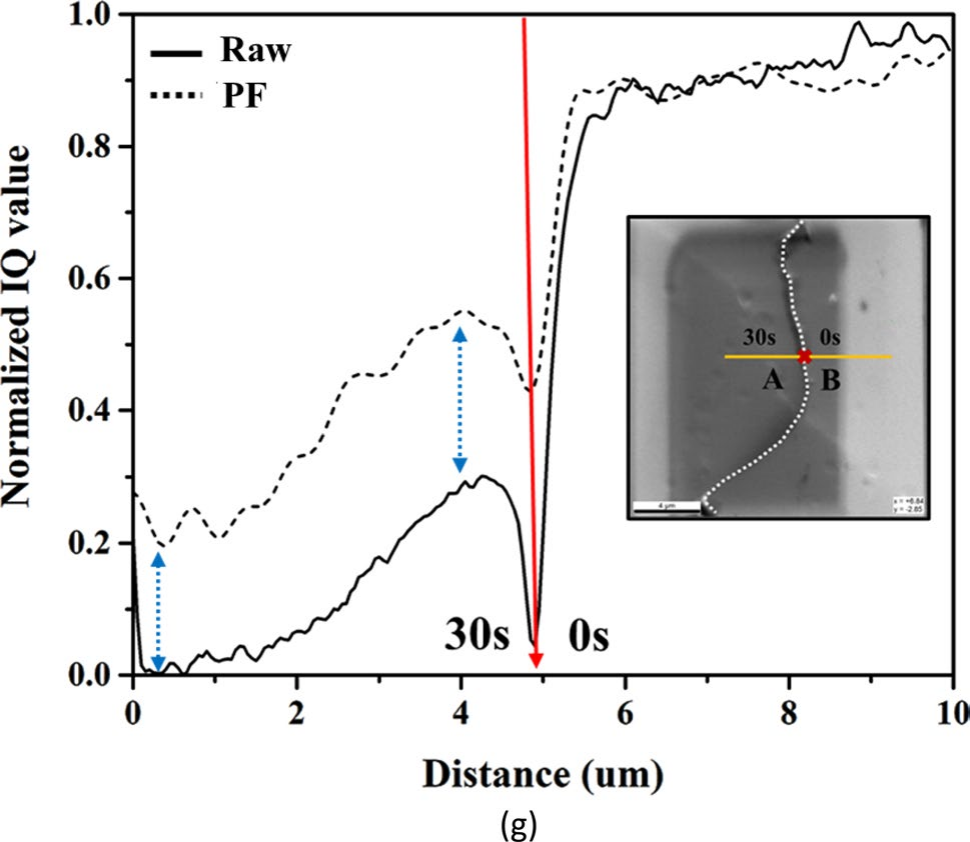
(**a**,**b**) Raw pattern, background-corrected patterns by (**c**,**d**) PA and (**e**,**f**) PF methods, where (**a**,**c**,**e**) are after 30 s Pt coating and (**b**,**e**,**f**) are without Pt coating at 5 kV. (**g**) Profiles of the normalized PQ values without and with coating obtained by EBSD line scanning, where the black solid and dash lines are without and with PF background correction, and the white dot line in the lower right image shows the boundary between coated and uncoated zone.

We used Ag bicrystals of A and B and coated a Pt layer only on the left side of the white dotted line in Fig. 6g, considering the effects of low accelerated voltage and conductive coating layer. Then, the raw patterns without and with coating were used to carry out background correction by PA and PF methods to compare the image quality between both methods. Figure 6a,b show the raw patterns with and without 30 s Pt coating layer on A and B grains. The figures show that the Kikuchi bands became blurred and diffused after Pt coating. Figure 6c,d and Fig. 6e,f display the results of Kikuchi patterns with PA and PF background correction method, respectively. The figures show that, for the case without coating, the PQ index does not have significant difference between PA and PF correction; the TenV index is 2.67 $\times$ 10^6^ for PA and 2.60 $\times$ 10^7^ for PF. In addition, the SSEQ index is 18.63 for PA and 4.95 for PF. After coating, the PQ, TenV, and SSEQ index values are 2319.3, 2.49 $\times$ 10^6^, and 37.55 for PA and 4789.3, 8.07 $\times$ 10^7^, and 6.07 for PF. The observations suggest that the contrast and sharpness of PF were improved compared with those of PA, and PF decreased noise.

We also used the normalized PQ based on Eq. (3) to quantify the image quality after PF correction and the normalization of PQ by the following^41^:

15 $${PQ}_{Normalized}=\frac{{PQ}_{value}-{PQ}_{min}}{P{Q}_{max}-{PQ}_{min}}$$

where ${PQ}_{max}$ and ${PQ}_{min}$ are the maximum and the minimum values of PQ, respectively. High PQ values indicate good pattern quality and can be better indexed after Hough transform^42^. The profile in Fig. 6g shows that the normalized PQ value significantly decreases after coating for both background corrections. Thus, the amorphous coating layer degrades the pattern quality. The difference in the normalized PQ value between PA and PF corrections reveals no significant change in the case without coating, whereas the normalized PQ value of PF correction in the case with coating is approximately twice that of PA correction. Hence, the results indicate that the PF background correction can improve the image quality of the coating layer at low accelerated voltage.

## Conclusions

The quality metrics of the diffraction patterns are evaluated using PQ, TenV, and SSEQ indices, which correspond to the clarity, high contrast, and high noise of the patterns, respectively. We propose a PF background correction method to extract background noise and diffraction pattern from raw diffraction patterns under low accelerated voltage or coated sample. After background correction, the PF method offers the best quality, with a PQ value of 24,062, a TenV value of 3.41 $\times$ 10^7^, and an SSEQ value of 18.92, for diffraction patterns obtained at 5 kV compared with the PA and PF methods. Thus, this proposed PF method can improve the quality of diffraction patterns in EBSD measurements at low accelerating voltages and with coating layer.

## Methods

### Acquisition of raw Kikuchi patterns

All the experimental Kikuchi patterns presented in this study were measured with a JEOL 7001 FE-SEM with a field emissions gun and an EDAX/TSL EBSD system equipped with a DigiView CCD camera and a phosphorescent screen with the diameter of 40 mm. The sample was tilted 70 degrees, and EBSD measurements were performed at a working distance of 15 mm. The parameter of Pt coating is 10 mA using sputter with 30 s. The raw Kikuchi patterns were captured using a 1 $\times$ 1 binning mode, and the gain value and exposure time were optimized to achieve high-quality patterns. In the line scan of EBSD experiment, the step size is 50 nm. Each raw Kikuchi pattern with a resolution of 1024 $\times$ 1024 pixels in 8-bit digitization was acquired, and the following image processing procedures were conducted using MATLAB.
